# Bronchoperitoneal and Enterocutaneous Fistula Development Following a Colorectal Anastomosis Leak

**DOI:** 10.7759/cureus.6277

**Published:** 2019-12-02

**Authors:** Peter A Ebeling, Jacob Malmquist, Katherine Beale, Deborah L Mueller, Jason Kempenich

**Affiliations:** 1 Surgery, University of Texas Health Science Center, San Antonio, USA; 2 Surgery, Univeristy of Texas Health Science Center, San Antonio, USA

**Keywords:** bronchoperitoneal fistula, enterocutaneous fistula, critical care

## Abstract

Bronchoperitoneal fistulas are rare but serious pathologies that pose numerous treatment challenges to physicians. There is usually a delay in diagnosis, and treatment recommendations are mainly derived from case reports. Here, we present an interesting case of a patient who developed a left bronchoperitoneal fistula and two subsequent enterocutaneous fistulas resulting from a massive intra-abdominal phlegmon eroding through the left diaphragm. The patient experienced numerous medical complications during his hospital stay and required multiple operations. However, 18 months after his initial post-operative complication, the bronchoperitoneal fistula has healed, and the patient has undergone successful intestinal reconstruction. This case highlights multiple uncommon disease processes and the treatment strategies used.

## Introduction

Bronchoperitoneal fistulas are rare entities that pose significant challenges to critical care and surgical teams. These fistulas most commonly occur due to erosion by a subdiaphragmatic abscess or mass and place the patient at significant risk for pneumonia, sepsis, acute respiratory distress syndrome (ARDS), and prolonged mechanical ventilation [1-3]. Although bronchoperitoneal and bronchopleural fistulas have been well described as complications after biliary surgery, their occurrence outside of this context is less common [4]. Bronchoperitoneal fistulas may heal with medical therapy alone, but surgery is sometimes required to evacuate an offending abscess or close off the thoracic cavity. Here, we present an interesting case of a patient who developed a bronchoperitoneal fistula and eventually two enterocutaneous fistulas following major abdominal surgery. 

## Case presentation

**Figure 1:**
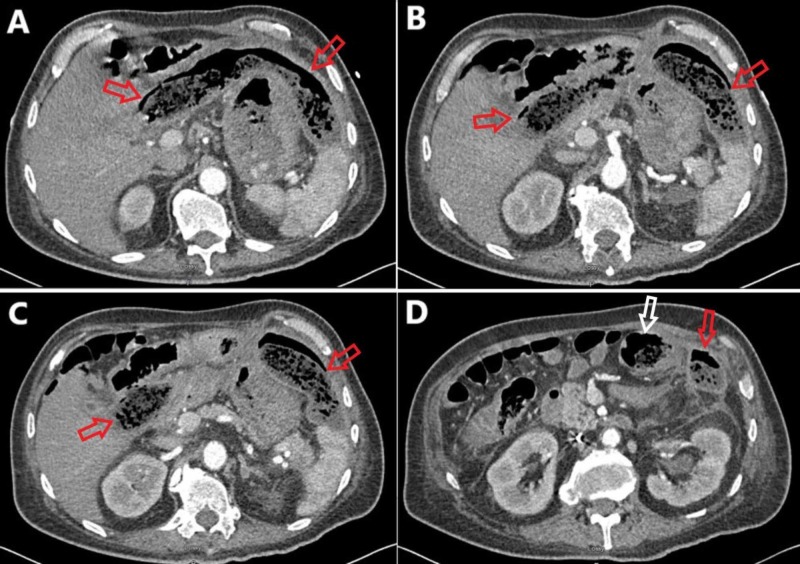
Contrasted computed tomography imaging of the abdomen and pelvis in the axial plane progressing from superior (A) to inferior (D). (A) A large, feculent phlegmon (red arrows) is apparent in the left upper quadrant anterior to the stomach and reaching the liver. (B) The phlegmon (red arrows) continues its inferior course in the abdomen. (C) The phlegmon spans into both the left and right upper abdominal quadrants (red arrows). (D) The phlegmon (red arrow) is in close proximity to the transverse colon (white arrow).

A 61-year-old man was transferred to the authors’ institution from another facility for the management of a suspected bronchoperitoneal fistula following an exploratory laparotomy to address a late complication from a prior segmental colectomy. The patient’s past medical history was significant for a near-obstructing sigmoid colon adenocarcinoma. This was treated with a laparoscopic converted to open-extended left hemicolectomy with colo-rectal anastomosis. His immediate post-operative course was unremarkable. He presented to the outside hospital three months after this index operation reporting abdominal pain and early satiety. Computed tomography (CT) imaging showed a large, walled-off collection of fluid and debris in the left upper abdominal quadrant (Figure 1). 

**Figure 2:**
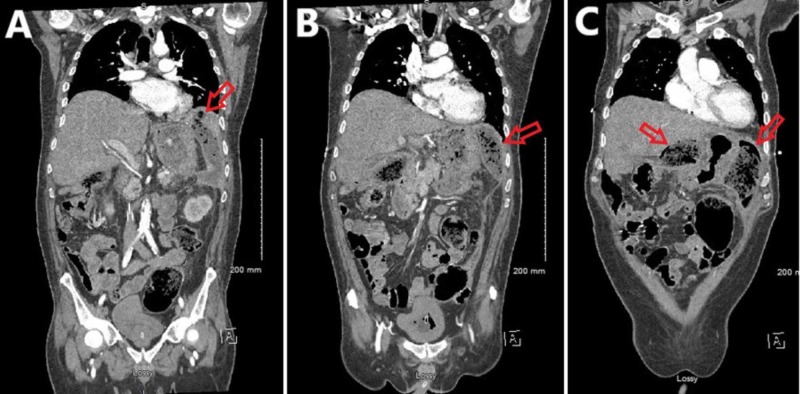
Contrasted computed tomography imaging of the abdomen and pelvis in the coronal plane progressing from posterior (A) to anterior (C). (A) The phlegmon (red arrow) is in close proximity to the left diaphragm. (B) The phlegmon extends inferiorly within the peritoneal cavity (red arrow). (C) The phlegmon is visible in the left upper abdominal quadrant and the mid-abdomen (red arrows).

The collection appeared to abut the left diaphragm and extended inferiorly and across the midline (Figure 2).

**Figure 3:**
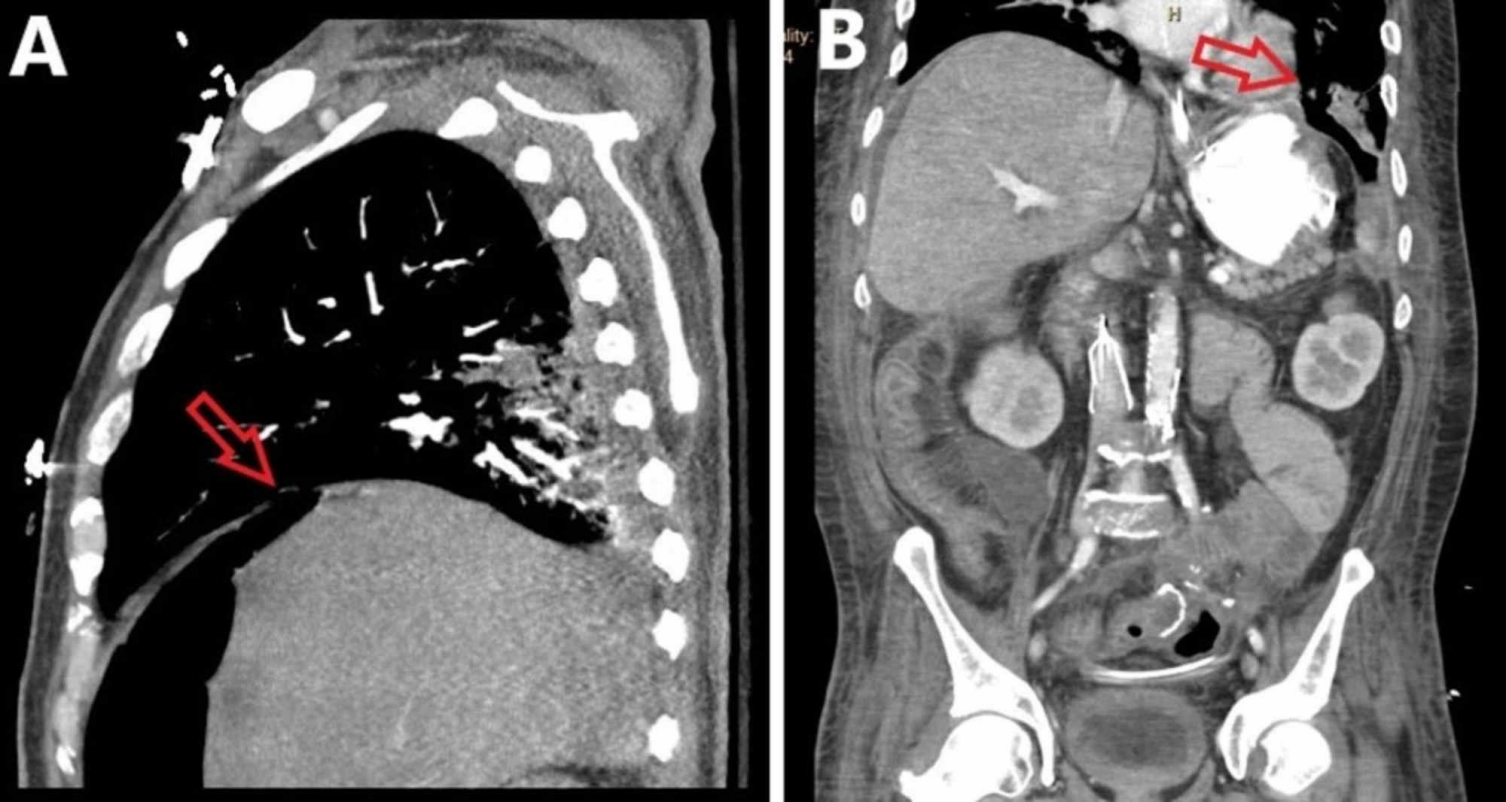
Contrasted computed tomography of the chest (A) and the abdomen and pelvis (B). (A) Sagittal plane computed tomography of the chest demonstrating a left bronchoperitoneal fistula (red arrow). (B) Coronal plane computed tomography of the abdomen and pelvis demonstrating a left bronchoperitoneal fistula (red arrow).

The patient subsequently underwent an exploratory laparotomy, evacuation of a feculent phlegmon, completion colectomy, and ileorectal anastomosis with diverting loop ileostomy creation. There was an inadvertent gastrostomy and two duodenostomies which were repaired primarily and buttressed with jejunal serosal patches. On post-operative day 2, the patient developed acute hypoxic respiratory failure requiring intubation and mechanical ventilation. On examination, air was heard escaping from the patient’s abdominal incision with every breath. The patient was taken for a CT scan of the chest, abdomen, and pelvis to assess for pneumonia or abdominal sepsis as a source of his deterioration. It demonstrated a left bronchoperitoneal fistula (Figure 3). 

The patient was then transferred to the authors’ institution for higher level of care and input from cardiothoracic surgeons. After he was resuscitated in the intensive care unit, the patient was taken back to the operating room to evaluate and control the bronchoperitoneal fistula. Upon entering the peritoneal cavity through the prior laparotomy incision, air was heard escaping into the abdomen coinciding with the patient’s ventilated breaths. The abdomen was irrigated with saline, and air bubbles were seen percolating through the left chest into the abdomen. The prior duodenostomies were re-buttressed with jejunal serosal patches. A feeding catheter jejunostomy (FCJ) was placed for enteral feeding access. Two large drains were placed in the left subdiaphragmatic space to control the fistula. Primary repair of the diaphragm defect was not attempted due to significant inflammation and immobility of the stomach. The patient was returned to the intensive care unit in stable condition. 

The abdominal drains were connected to a Pleur-evac chest drainage unit and placed to wall suction. An air leak was noted, but it resolved by post-operative day 5 despite continuous positive pressure ventilation. The drains were disconnected from suction approximately two weeks post-operatively. However, several days later air was noted escaping from the abdominal incision. Therefore, the drains were placed back to suction, and evidence of pneumoperitoneum was resolved. 

Approximately one month into his hospital stay, the patient developed an acute pulmonary embolus and was initiated on therapeutic anticoagulation. He subsequently experienced an intra-abdominal hemorrhage. The abdomen was re-explored in the operating room, but no bleeding source was identified. At this time, there was no audible air passing into the abdomen from the left chest. However, two enterocutaneous fistulas were noted, one originating from the gastroduodenal junction and the other involving the jejunum. Additionally, one of the patient’s prior duodenotomy repairs had dehisced. Given that this duodenal injury failed to heal after two earlier repairs, a robnel drain was placed retrograde into the duodenum through this duodenotomy. Malecot drains were placed in each fistula and externalized to serve as one controlled fistula. A large ostomy appliance was fashioned to encompass much of the patient’s abdomen. The fistulas were managed with maximum medical therapy including nil per os (NPO) status and total parenteral nutrition (TPN).The patient spent 91 days in the hospital. He experienced multiple medical complications, including respiratory failure requiring tracheostomy, fungemia, and malnutrition. He was discharged to home with home health services to assist with TPN infusions.

The patient’s nutritional status was optimized while on TPN, and fistulous output was managed with anti-secretory medications. Despite these interventions, the fistulas continued to drain approximately two liters of fluid per day. He required frequent intravenous fluid replacements, and his quality of life was greatly affected. Eventually, the patient was offered a fistula takedown.

**Figure 4:**
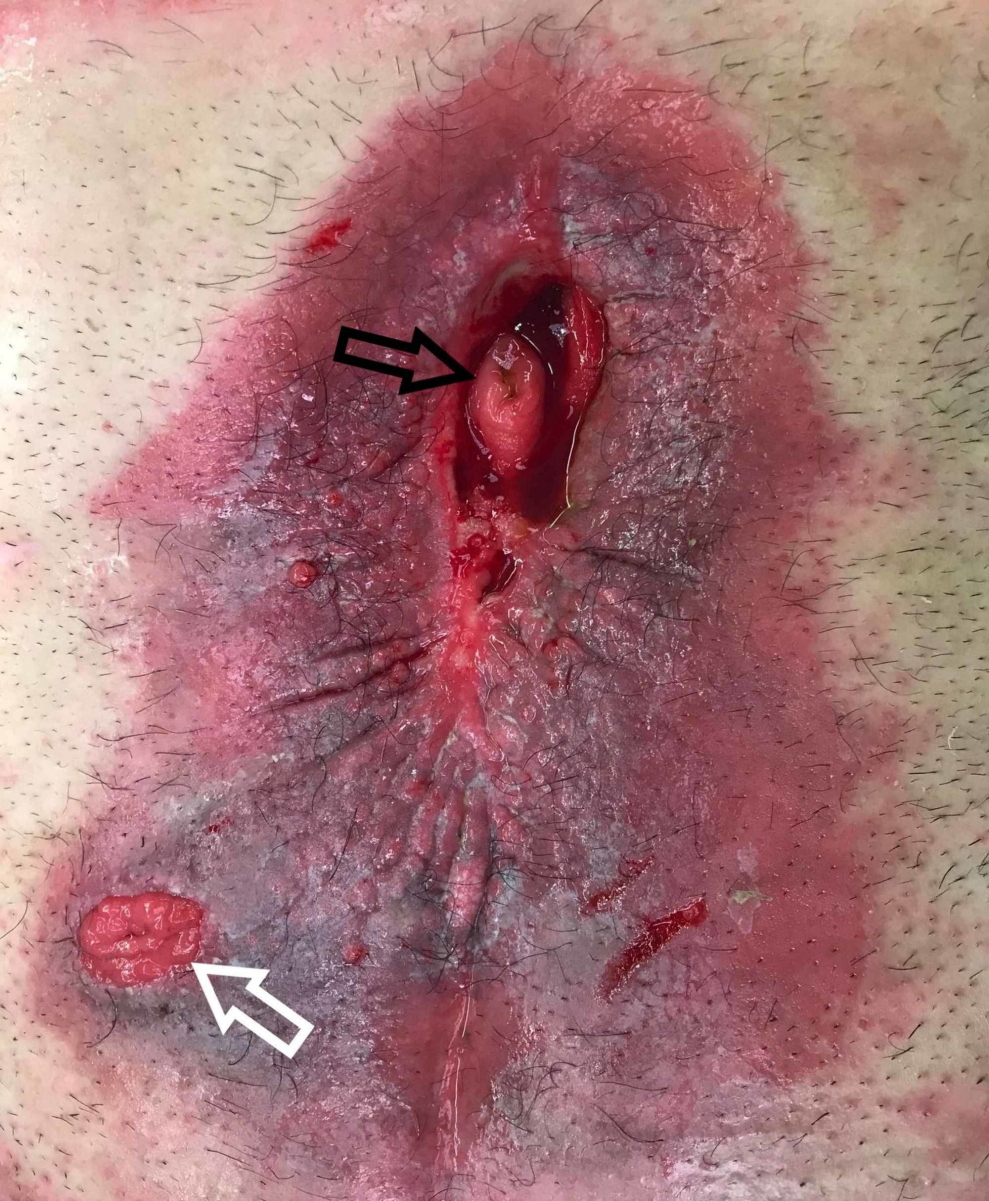
Enterocutaneous fistulas in the midline abdominal wound, including the jejunal fistula and the gastroduodenal fistula (black arrow). The diverting loop ileostomy is in the left lower quadrant (white arrow).

Approximately one year after discharge from the hospital, the patient returned for enterocutaneous fistula takedown, FCJ placement, and complex abdominal wall reconstruction. At this point, we had determined the patient had one gastroduodenal fistula as well as a jejunal fistula. The enterocutaneous fistulas on the day of the operation are shown in Figure 4. We performed a primary closure transversely of the gastroduodenal fistula in a Heineke-Mikulicz pyloroplasty fashion. The jejunal fistula was resected. We left the loop ileostomy in place to avoid a second anastomosis. Bilateral separation of components was necessary to close the abdomen. We also placed an absorbable mesh in an overlay fashion.

The patient’s post-operative course was notable for candidemia and bacteremia, likely secondary to his peripherally inserted central catheter which was used for home TPN infusions. Infectious disease specialists were consulted, and the patient completed appropriate antimicrobial therapy. He was discharged to home after 26 days in the hospital tolerating a normal diet and nocturnal feeds through the FCJ. At his two-week follow up-appointment, the patient demonstrated adequate weight gain, and the FCJ was removed.

## Discussion

Fistulas between the peritoneal cavity and bronchi are rare. Bronchoperitoneal fistulas most commonly occur when an inflammatory process, such as a subdiaphragmatic abscess or mass, erodes from the peritoneal cavity through the diaphragm into the bronchi [1]. There is one report of a lung abscess eroding through the diaphragm in a cephalo-caudal direction resulting in a fistula which required operative intervention [3]. Saliba et al. detailed a left bronchoperitoneal fistula resulting from an abscess cavity eroding through the diaphragm which developed as a result of delayed leak following sleeve gastrectomy [5]. The patient ultimately required a laparoscopic esophagojejunostomy with gastric exclusion and primary repair of the diaphragm defect. Additionally, iatrogenic injuries from biliary procedures and even retained surgical drains have been noted to result in bronchoperitoneal fistulas [2,4]. 

Bronchoperitoneal fistulas are potentially life-threatening. Patients are at high risk for pneumonia, sepsis, ARDS, and prolonged mechanical ventilation requiring tracheostomy. Aggressive strategies to minimize infectious complications should be initiated, including intravenous antibiotics to empirically cover enteric and respiratory flora. Ventilator management in this patient cohort is particularly challenging. There are several reports of novel ventilator strategies used to treat patients with bronchopleural fistulas, which may be applicable to bronchoperitoneal fistulas [2,4,6]. In general, the goal of ventilation therapy is to minimize air flow across the fistula. Spontaneous breathing is preferred as it provides a decreased transpulmonary pressure gradient with less leak. Unfortunately, most patients are usually unable to generate sufficient respiratory effort due to their overall disease burden. If mechanical ventilation is necessary, then decreased air flow through the fistula can be achieved with a high-frequency, low-tidal volume approach or by equalization of the airway pressure and the interpleural pressure [7]. Multiple cases utilized high-frequency oscillatory ventilation to minimize air leaks, barotrauma, and peak airway pressure [2,8]. This approach may facilitate healing in both bronchoperitoneal and bronchopleural fistulas. Despite the many challenges associated with bronchoperitoneal fistulas, most patients from the reviewed case reports achieve adequate recovery one to two years after treatment. It is notable the patient in this report experienced healing of the fistula despite being on conventional ventilation modes.

Surgery’s role in managing bronchoperitoneal fistulas remains poorly defined. Based on our experience and review of the available literature, surgery should be considered to intervene on an extrinsic mass and place appropriately sized drains to minimize flow across the fistula. As most bronchoperitoneal fistulas originate from a benign or malignant mass eroding through the diaphragm, it would be prudent to remove the offending agent. Four of the case reports reviewed ultimately required operative management to obtain source control, debride non-viable tissue, or repair the diaphragm [1-3,5]. Surgical treatment also permits inspecting the abdomen for additional injuries. It is also notable that there were concomitant small bowel injuries at the time of laparotomy in our case and in at least one other report [2]. As this case demonstrates, repairing the diaphragm is not always necessary to promote fistula healing. However, we contend leaving a diaphragmatic defect warrants placing drains to minimize flow across the fistula. 

Drain management warrants particular attention, as it may play a key role in healing a bronchoperitoneal fistula. Drains reduce air flow across the fistula and limit pneumoperitoneum. This is especially important if there are concomitant bowel injuries with recent or tenuous repairs. Air rushing into the peritoneal space under positive pressure may directly disrupt bowel repairs or impair intestinal healing by elevating intra-abdominal pressure. Even short-term increased intra-abdominal pressure has been implicated in impaired bowel healing, bowel tissue hypoxia, and anastomotic failure in animal models [9,10]. It is noteworthy that the patient in this report showed evidence of air escaping from the abdominal incision only after the intra-peritoneal drains were placed to water seal. Subsequently, the surgical team discovered the duodenotomy repair had failed. There are no trials and only limited case reports from which to draw conclusions regarding how long to leave these drains connected to suction. However, our experience would suggest that leaving drains connected to suction for a longer duration may be beneficial. As post-operative intestinal leaks are extremely uncommon at six weeks, we would recommend leaving drains connected to wall suction for eight weeks in cases with concomitant bowel injuries or repairs [11]. 

## Conclusions

Bronchoperitoneal and enterocutaneous fistulas are uncommon pathologies. This case demonstrates treatment options for both types of fistulas. Surgeons and critical care physicians should be aware that bronchoperitoneal fistulas are challenging to promptly diagnose. These patients may require novel ventilator management strategies and surgical treatment. 
